# The Royal Hants County Hospital, Winchester

**Published:** 1892-07-09

**Authors:** 


### WITHIN THE HOSPITALS.

### IV.—THE ROYAL HANTS COUNTY HOSPITAL, WINCHESTER.

This hospital is situated on a hill, and commands a fine view of Winchester and the district. The site, when reached, leaves little to be desired, and it was delightful to notice the abundance of wild flowers to be met with on one side of the hospital, where the grounds have been allowed to run wild. The hill which has to be ascended is, however, a great drawback to patients attending the hospital, and to obviate this the Committee, with the aid of a wealthy Governor, have organised an omnibus service to and from the hospital. This is one of many instances of the thoughtful care for the welfare of the patients displayed by the management. The view from the terrace on a fine day is superb, and below, on the side of the hill, there are extensive kitchen gardens, while the terrace itself is laid out in grass, which two tame gulls have almost to themselves. This is an age in which art is supposed to imitate nature so accurately as to make it difficult for short-sighted people to easily distinguish one from the other. Thus your Commissioner mistook the two gulls which he noticed standing together with a plaster-like rigidity for plaster-casts instead of birds. The comical aspect of these two gulls when seen was very amusing, and the effect was deepened when we paid them a visit on the lawn, where they strutted about with military precision and the utmost self-possession. These gulls had been there for some years, and appeared to be as pleased with themselves as they are pleasant to the patients and all beholders. They would form excellent models for Mr. H. Stacy Marks, R.A., who would rejoice, we are certain, to serve them up as "Two Intelligent Out-patients."

#### The Hospital Buildings.

The present buildings, erected in 1867, are an excellent example of the straight pavilion type. The men's wards are situated at right angles to each other, and are divided by a central staircase and the administration buildings. These wards are excellently proportioned, but we noticed that two beds, and one especially, at the far end of each ward ought to be taken down, because it is impossible to give them the indispensable minimum of air, light, and floor space. We ascertained that cases of sore throat had manifested themselves in these beds, and we hope the medical staff, remembering that the hospital always contains more beds than there is a demand for, will order them to be removed from the wards. In all other respects the wards are excellent specimens of their type. Indeed, it is not too much to say that, having regard to the date when they were built, they constitute one of the best examples of a pavilion ward. Height, width, light, and floor space, and the disconnection of lavatories and bath-rooms, leave little, if anything, to be desired. Another fact worthy of note is the circumstance that the Parian cement, except in one ward, has stood the test of time at this institution. The walls have never been painted, and they are as good and sound to-day as they were when originally opened. This is the first instance in our experience where Parian cement has behaved itself properly, and we note the circumstance because it may tend to prove that good workmanship and good material may lessen, if they do not entirely remove, the objections which have arisen with reference to this material. It is a feature of the Royal County Hospital that each of the female patients has to wear a regulation white cap, with a blue or a black ribbon, as may be preferred. There was an abundance of flowers in the wards; the nurses took a very great interest in their ferns and flowers, and all the patients appeared to be happy and comfortable. Painted iron baths are *de rigueur* at this institution, but we must once more state our opinion that none but porcelain baths are suitable to hospital purposes. Possibly the Committee may see their way at some future time to replace the whole present baths by Rufford's or Cliffe's. This expenditure would give them a great deal of satisfaction. With new slop-sinks and fittings in the lavatories and ward kitchens the hospital would be almost up to date.

#### The Accommodation for Children.

It has long been the practice at this institution to mix children and adults in the general wards. Our experience teaches that this system is neither good for the patients nor good for the children. We were assured that the Committee had considered the question more than once and that they had resolved not to change it because the nurses when questioned agreed that it was pleasant to have a certain number of children to look after in addition to the adults. Be this as it may, we would strongly urge the Committee to again consider the matter, and to at once make arrangements to fit up one of the two central wards as a children's ward, and to remove all the children to it. Not only would this alteration greatly add to the comfort of the patients as well as of the little ones, but it would form a most popular feature of the institution, and so tend to increase the income to an extent which the Committee can hardly realise until they have given the plan a fair trial.

#### The Out-Patient Department and the Kitchens.

Having regard to the fact that the principal buildings were erected a quarter of a century ago, and that the number of out-patients is relatively few, it would be unfair to severely criticise the present out-patient department. This is situated in the basement beneath one of the large wards, an arrangement which would not be sanctioned nowadays in a new hospital building. The kitchen is also situated in the basement, and these two sources of possible unpleasantness might usefully be abolished whenever funds enable the Committee to provide new quarters for their accommodation.

#### General.

Although the main staircase is excellently proportioned and so constructed as to afford the maximum of facility for taking patients up and down stairs, the passages of communication which connect the two main wards on each floor are too narrow and insufficiently lighted. This is relatively a minor point, but it has to be mentioned in considering the planning of the whole building. A large chapel is situated at the top of the hospital, which occupies a great amount of space, and struck us as being altogether too large for the size of the hospital. It is commendable from the fact that the heating arrangements are adequate, a circumstance which is too often overlooked in connection with hospital chapels which have to be used by people in delicate health. In all other respects the buildings are excellent, and their present condition reflects credit on all departments.

